# Short term outcomes and complications of distal ulnar ostectomy in 23 juvenile dogs with carpal valgus secondary to discordant radial-ulnar physeal growth

**DOI:** 10.3389/fvets.2022.971527

**Published:** 2022-09-09

**Authors:** Scott Christopher

**Affiliations:** Sage Veterinary Centers, Campbell, CA, United States

**Keywords:** limb deformity, ostectomy, canine, orthopedics, surgery

## Abstract

**Objective:**

The goal of this study was to report short term clinical and radiographic outcomes after distal ulnar ostectomy in dogs with carpal valgus due to discordant radial-ulnar growth.

**Study design:**

Retrospective case study.

**Sample group:**

Client owned dogs under 1 year of age with carpal valgus and open distal radial physes pre-operatively.

**Methods:**

Medical records from four veterinary referral centers were searched from January 1, 2015 to January 1, 2022 for juvenile dogs that had been treated with distal ulnar ostectomy for carpal valgus due to premature closure of the distal ulnar physis. Patients were excluded if they were skeletally mature at the time of ostectomy; medical records were incomplete; radial physis was closed at surgery; or definitive corrective osteotomy was performed. Radiographs were evaluated pre-operatively and for short term follow up at ~8 weeks. Complications and short term clinical outcomes were evaluated also.

**Results:**

31 limbs from 23 dogs were evaluated. Patients ranged from 4 to 10.8 months of age. All dogs presented for visible carpal valgus and varying degrees of thoracic limb lameness. Sixty-four percent of patients showed resolution of lameness while an additional 13% showed an improvement in clinical lameness without complete resolution. Complications were seen in 32% of patients with 70% percent of those being minor, bandage related complications. Radiographically, 38% of limbs showed bridging callus formation of the ostectomy at an average of 7.5 weeks post operatively and 75% percent of patients with elbow incongruity improved radiographically. There was no significant difference in radial joint angles pre-operatively and at the time of follow up.

**Conclusion:**

Distal ulnar ostectomy ameliorates lameness in juvenile dogs with premature distal ulnar physeal closure and shows lack of progression of distal carpal valgus deformity, but does not improve joint angulation.

**Clinical significance:**

Distal ulnar ostectomy is associated with mild bandage-related complications and halting of progressive limb deformity within the time frame evaluated, and should therefore be considered a treatment for premature closure of the distal ulnar physis. It does not lead to deformity correction at 8 weeks following surgery but is associated with improved elbow congruity.

## Introduction

Physeal injuries were first described in human children in 1963 by Salter and Harris (1), and their description scheme has been used in veterinary medicine. Physeal injury can result in a loss of longitudinal growth or asymmetric growth of the affected bone. In limb segments with paired bones such as the radius and ulna, abnormal growth of the affected bone can subsequently influence the growth of the unaffected bone, owing to the strong soft tissue attachments between the bones (2, 3). The bone involved, extent of physeal injury, patient age, and remaining growth potential determine the extent of growth discrepancies and angular limb deformity formation. In canine patients, the conical distal ulnar physis, which is responsible for 85–100% of ulnar growth (4), is susceptible to Salter Harris type V (crushing) injury, which can result in halting of distal ulnar growth and subsequent angulation of the distal radius and ulna, even if the radius itself is not injured. Specifically, distal ulnar physeal injury can lead to distal antebrachial valgus, torsion, and procurvatum and elbow incongruity (5). In addition to physeal injury, carpal valgus may be caused by developmental or hereditary abnormalities. Abnormalities in endochondral ossification can lead to altered physeal growth (5, 6). Chondrodystrophy and chondrodysplasia are genetic defects in fibroblast growth factor (FGF4a) (7, 8) that result in similar limb deformities (9, 10) to those seen in dogs with premature closure of the distal physis. However, chondrodystrophic dogs are more likely than other dogs to have more complex, biapical limb deformities, often with elbow joint incongruity (11).

While there are a variety of causes of angular limb deformities (ALDs), the progression without intervention is often inevitable. The progression of ALDs during development is thought to be a vicious cycle of asymmetric growth cause by an alteration in the application of mechanical forces across the epiphyseal growth plate as well as bone remodeling. Bone growth is dependent on many factors ranging from patient age, patient size, nutrition, hormones and many more but one of the most important factors are the external stresses applied to the bone which are described by Wolff's law and the Hueter-Volkmann law. Wolff's law describes adaptations of the bone induced by increased mechanical load leading to increased bone density. It is not often thought to be a primary factor in epiphyseal growth. The Hueter-Volkmann law describes growth principles around joints and joint deformation in which there are alterations in longitudinal growth rates in response to tensile and compressive loads. Increasing compressive load slows epiphyseal growth while increasing tensile forces accelerates growth. This has been shown to be dependent on the overall magnitude of the load rather than solely the average load (12). True longitudinal growth appears to rely on static and to some degree intermittent loading (13, 14) while bone remodeling relies more on intermittent loading (15). However, there is concern that high compressive dynamic loads may also significantly alter growth plate development (16–18). In most cases, these forces ultimately are thought to be reversible if the proper counter forces are applied (19).

Elbow dysplasia is a complex developmental disease in dogs influenced by both environmental and hereditary components. It consists of conditions such as fragmented medial coronoid process, ununited anconeal process, and elbow incongruence. Elbow incongruence alone is a source of lameness and is also thought to result in a predisposition to progressive elbow joint osteoarthritis (20, 21). As research on these conditions progresses, discordant growth between the radius, ulna, and humerus amongst other factors become the focus as a causative agent. These conditions persist after disparate bone growth (22, 23) leading to joint trauma and subsequent degenerative joint disease. In the case of ununited anconeal process, Sjöström et al. have reported that removing the disparate growth through an ulnar ostectomy allowed 21 of 22 ununited anconeal processes to fuse. They used an additional case to document the progressive development of the ununited anconeal process (24). Fragmented medial coronoid process is caused by progressive microfracture of the underlying subchondral bone with or without cartilage fissures (25), and is believed to arise from overloading of the medial compartment due to disparate growth (26). Although numerous factors must be considered in the underlying pathogenesis of elbow dysplasia, joint incongruence as a sole condition or predisposing factor continues to be considered a major cause of and basis for treatment options.

Whether secondary to trauma, developmental abnormalities, or defects in endochondral ossification, surgical treatment of radial ulnar discordant growth in juvenile dogs includes removal of a distal ulnar bone segment with or without additional manipulation of the distal radial growth plate (27–29). Distal ulnar ostectomy allows the radius to grow without an influence from the shortened ulna, thus preventing or resolving mild elbow incongruency and minimizing the progression of antebrachial deformity although definitive corrective ostectomy may be necessary. Although this procedure is commonly performed, no recent studies have evaluated the complications and outcomes associated with the distal ulnar ostectomy procedure alone as a treatment for premature closure of the distal ulnar physis in juvenile dogs.

Previous studies evaluating premature physeal closure have described distal ulnar ostectomy in a larger group including animals receiving corrective osteotomies after physis closure. Ramadan et al., in 1978, evaluated 58 dogs with premature ulnar physeal closure. Only eight of those patients were treated with an ulnar ostectomy, and all patients were also treated with radial physeal stapling (5). Morgan et al. (30), evaluated 24 dogs, 12 of which were treated partial ulnar ostectomies; each showed improvement in frontal limb alignment, and 60% had good or excellent outcomes.

Herein, on the basis of early literature, distal ulnar ostectomy was hypothesized to result in improved joint orientation lines caused by abnormal distal ulnar physeal growth regardless of premature ostectomy bridging in dogs with distal antebrachial valgus and procurvatum, without significant torsional deformity. A secondary aim of this study was to outline the short-term complications and radiographic changes associated with this procedure.

## Materials and methods

### Case evaluation

**Figure 1:**
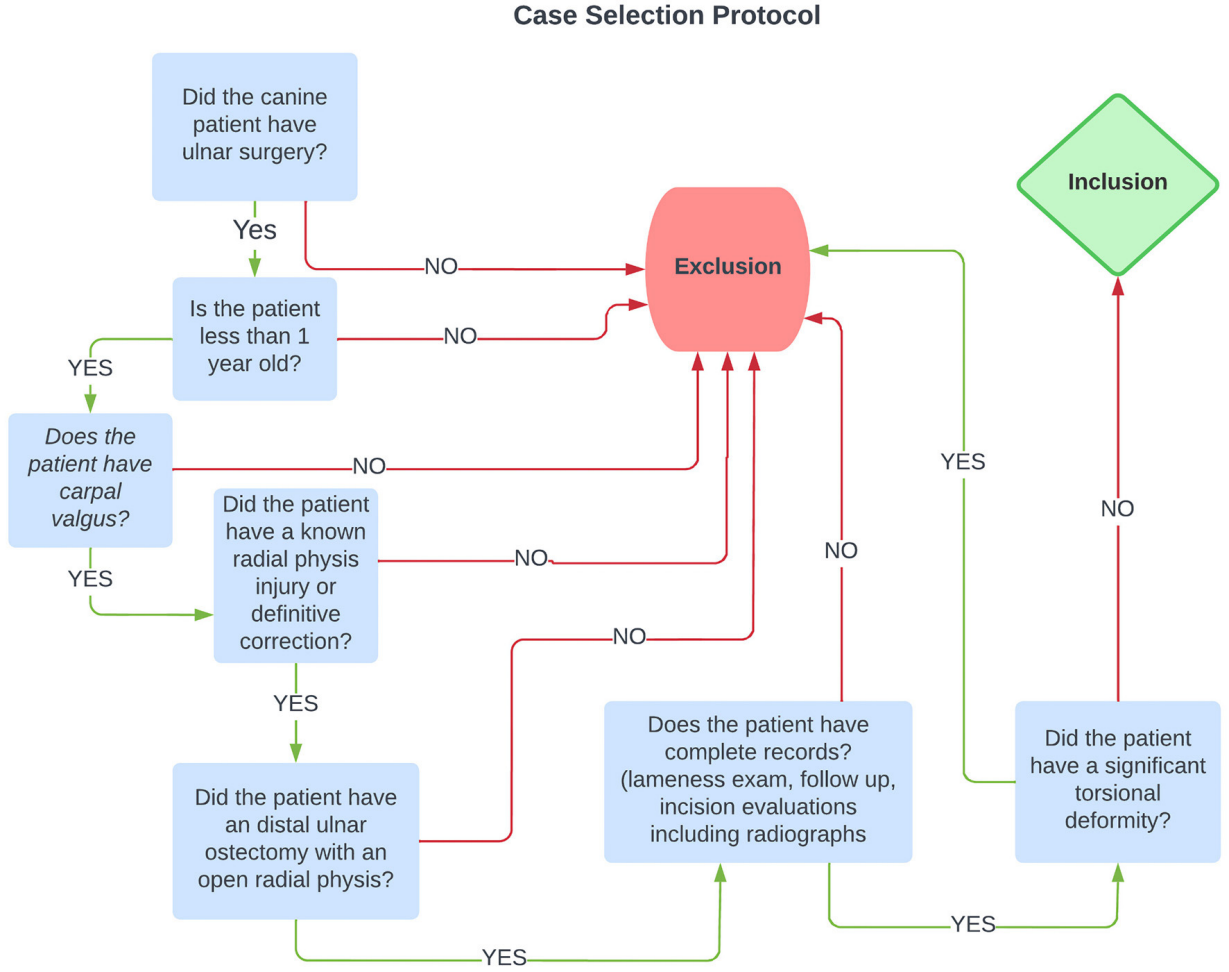
The case selection process, as described, is shown in flow chart form.

Medical records were searched for all dogs that underwent an ulnar ostectomy of any type from January 1, 2017 to January 1, 2022. Medical records were searched electronically for all patients who had radius or ulna surgery. All canine patients who had ulnar surgery were first evaluated. All dogs >1 year old at the time of surgery were excluded. Patients were then included if they had carpal valgus but excluded if they had known radial growth plate injury and if there was a definitive corrective osteotomy performed at any time during the study period or prior to radial physeal closure. Dogs were included if an ostectomy in the distal half of the ulna was performed; the radial physis was open at the time of ulnar ostectomy; and records included lameness exam, pre-operative and post-operative evaluations, incision evaluations with or without bandage change and follow up evaluation and imaging at least 6 weeks post-surgery were available. The presence of phone communication between surgery and suture removal and/or bandage change appointment was a requirement to increase the likelihood of identifying minor complications. Dogs were finally included if there was no significant torsional component to the deformity before surgical treatment. This process is outlined in Figure 1. Signalment information, including breed, sex, age, and weight at the time of surgery, was recorded. Lameness evaluation was measured on a subjective scale of 0–5 as shown in Table 1. A chondrodystrophic breed was considered one genetically predisposed to chondrodystrophy or chondrodysplasia, on the basis of FGF-4 genetic mutations within the breed (7, 8) including breeds such as the Basset hound, Cardigan and Pembroke Welsh corgi, dachshund, and French bulldog amongst others. However, mixed breed dogs presenting with bilateral forelimb deformities without evidence of traumatic physeal injury were considered chondrodystrophic for statistical purposes as Chondrodystrophy has been described in a large range of breeds (10, 31). Chondrodystrophic dogs were included in this study as they are one of the more common patients and in the absence of genetic testing for the FGF4 mutation is based off of clinical evaluation.

**Table 1:** Subjective lameness evaluation.

**Numeric grade**	**Description**
0	No lameness
1	Mild lameness, requiring trained eye to see
2	Moderate lameness with normal stride length with partial weight bearing
3	Moderate lameness with shortened stride length and partial weight bearing
4	Severe lameness with toe touching weight bearing lameness
5	Non-weight bearing lameness

The operated limb, ostectomy size, use of a fat graft, location of the ostectomy, total anesthesia time, and use of a post-operative bandage were noted. Dogs with unilateral and bilateral disease were included. The effect of unilateral disease vs. bilateral disease will be assessed in statistical analysis to determine if there is a difference in clinical outcomes. The total anesthesia time for bilateral procedures was analyzed independently of unilateral procedures if the both forelimb procedures were performed simultaneously, and all other criteria were analyzed independently. The use of pre-operative and continued non-steroidal anti-inflammatories (NSAIDs) was evaluated. The frequency of NSAID usage was not evaluated. The presence of post-operative complications, clinical improvement from initial examination to follow up at 8 weeks, and any recorded treatment for post-operative complications were also noted. Post-operative complications were classified as minor, major, and catastrophic, as previously described (32). Complications were also characterized on the basis of being bandage related, which are those complications that would not be expected to be seen in the absence of a modified Robert-Jones bandage. Furthermore, the exact complication was recorded including interdigital dermatitis and skin wounds unrelated to the surgical incision for example.

### Surgical procedure

The patient was placed in dorsal recumbency with the limb four quarter draped and the distal limb wrapped in sterile vetwrap. A lateral approach was made to the distal ulnar physis using a 10 blade. Hemostasis was maintained using monopolar electrocautery. The subcutaneous tissue was bluntly dissected to expose the distal ulnar physis. Retraction for surgical exposure was maintained using gelpi retractors. A Hohmann retractor was placed between the ulna and radius to ensure iatrogenic damage to the radius did not occur. Exact ostectomy size and location varied from surgeon to surgeon and was quantified in the surgical report and location and percentage of limb length was evaluated radiographically. The ostectomy and periosteum were removed en bloc. The surgical site was lavaged with sterile saline. The intradermal layer of skin was closed with an absorbable monofilament suture followed by a non-absorbable skin suture if an external suture layer was placed.

### Radiographic evaluation

Pre-operative, immediate post-operative, and follow up radiographs were evaluated for radiographic healing, frontal and sagittal plane distal radial angulation, distal radial physeal closure, relative ulnar ostectomy length, and elbow congruity. Follow up radiographs were performed at ~8 weeks post-operatively. Torsional deformity in the frontal plane used for exclusion criteria was evaluated subjectively because of the lack of appropriate guidelines for evaluation of radial torsion on the basis of radiographs. A closed physis was defined as the absence of a clearly defined radiolucent line at the ossification center. Relative ulnar ostectomy length (%) was defined as the ulnar ostectomy gap length divided by the overall ulnar length in a lateral post-operative radiograph. Degenerative joint disease was defined as the presence of osteophytosis on the radial head or anconeal process (33). Ulnar notch sclerosis was not used because of the poor reliability of the assessment (34). Degenerative joint disease in the carpus was defined by the presence of osteophytes or enthesophytes in the carpal joint or subjectively increased joint effusion (33). Elbow incongruity was defined as a >2 mm radioulnar step, which has been described as a moderate incongruity (35) on the lateral radiographic projection, owing to the sensitivity in radiographic evaluation of incongruence (36, 37). The measurement of a radioulnar step was defined as the distance between the radial head and the lateral coronoid process (38). Humeroulnar incongruency was not able to be evaluated, because of the specific views necessary to obtain accurate and repeatable measurements (38). Bridging callus was defined as the presence of bony callus crossing the ostectomy site with at least three cortices of bone.

**Figure 2:**
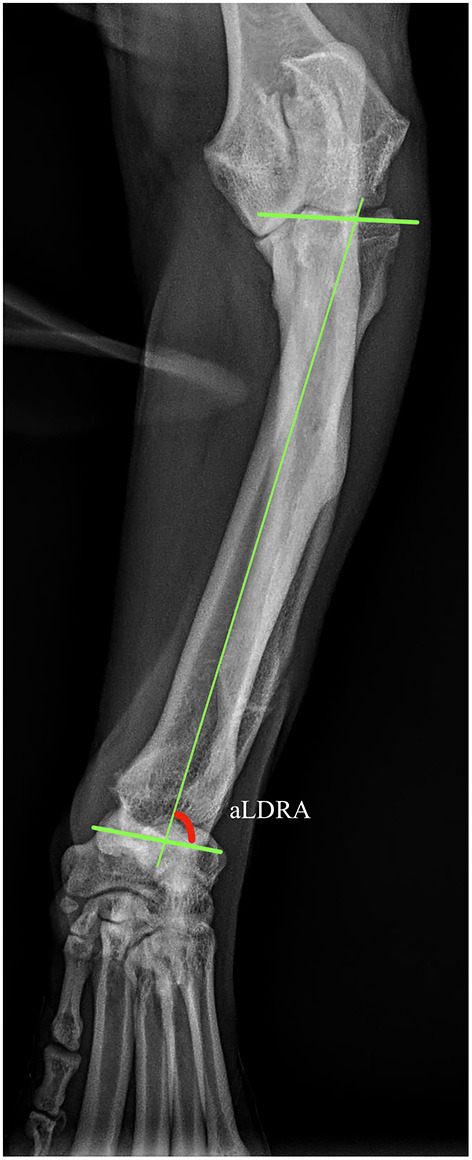
Frontal plane joint orientation angles evaluated in a representative dorsal palmar projection.

**Figure 3:**
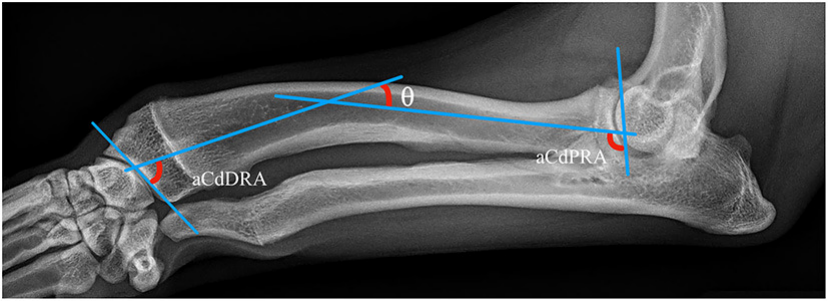
Sagittal plane joint orientation angles evaluated in a representative lateral projection.

Well positioned radiographs were required for measurement of angulation and elbow congruity. An acceptable craniocaudal antebrachial radiograph was defined by olecranon superimposition over the midportion of the humeral condyle in the cranial caudal projection, and an acceptable lateral antebrachial radiograph was defined by overlapping of the condyles. Patients with inappropriately positioned radiographs or significant torsion were excluded from joint measurements. All measurements were collected by a single blinded observer. For blinding, the DICOM images were calibrated and exported to jpg files using vPOP v 2.5.3 software (Vetsos Education ltd, Shrewsbury, UK). The ulna was digitally removed from the images with Pixelmator Pro (Pixelmator, Vilnius, LT) before measurements were performed. All radiographic measurements were performed in vPOP veterinary pre-operative planning software. These angles were compared pre-operatively and at the time of follow-up. Joint orientation lines were defined as previously described (39). In the frontal plane (Figure 2), the anatomic axis was drawn with a best fit line bisecting the radius, and the anatomic lateral distal radial angle (aLDRA) was measured (39). In the sagittal plane (Figure 3), the proximal and distal anatomic axes were defined individually by using a best fit line that bisected the respective segments of the bone to accommodate the natural procurvatum of the radius. The reference points are connected in both halves of the bone to form the two anatomic axes whose intersection (θ) is within the cortical confines of the bone and measurable. The anatomic caudal proximal radial angle (aCdPRA) and the anatomic caudal distal radial angle (aCdDRA) were measured using the intersection of this line and the joint orientation lines. The joint orientation lines are lines drawn from the proximocranial aspect of the radial head to the proximocaudal aspect for the proximal joint surface and the distocranial aspect of the radial articular surface to the distocaudal aspect of the radial surface for the distal orientation line. Procurvatum was calculated by using the sum of the proximal and distal joint orientation angles and the difference between segmental anatomic axes (θ): (90° – aCdPRA) + (90° – aCdDRA) + θ(39–41).

### Statistics

All statistics were evaluated using SPSS v28.0.1 statistical software (IBM SPSS, Chicago, IL). Descriptive statistics and frequencies were used to evaluate signalment, surgical times, and complications. Frontal and sagittal plane joint orientation angles from initial radiographs and follow up radiographs at 8 weeks were compared with a paired *t*-test. Shapiro-Wilk tests were used to evaluate normality. One-way ANOVA tests were used to evaluate the effect of anesthesia time on complications. Kruskal-Wallis tests were used to determine if ostectomy size played any role in complication type. Repeated measures ANOVA tests were used to evaluate the effect of age at the time of surgery on change in joint orientation angles. A significance level of 0.05 was used for all testing.

## Results

Fifty-one dogs with ulnar ostectomies performed between January 1, 2017 and January 1, 2022 were identified. Twenty-eight dogs were excluded because of incomplete medical records or concurrent deformity correction. Eight dogs had bilateral procedures performed, thus resulting in a total of 31 limbs evaluated. Bilateral procedures, when compared to unilateral procedures, did not carry a statistical difference in any of the categories so the limbs were treated independently in all analysis. Eight dogs were considered chondrodystrophic. Table 2 shows the weight and age of treated dogs. Breed, gender, and side treated are documented in Table 3. Follow up was intended for 8 weeks but ranged from 6 to 9 weeks, with an average follow up of 7.55 ± 1.21 weeks.

**Table 2:** Mean and standard deviations for age and weight of the treated animals.

	**Mean**	**SD**
Age (months)	8.14	1.82
Weight (kg)	17.37	6.95

**Table 3:** Frequencies for sex, treated limb, and breeds.

**Variable**	***N***	**%**
**Sex**		
M	8	25.8
MC	14	45.1
F	2	6.5
FS	7	22.6
**Side**		
Left	19	61.3
Right	12	38.7
**Breed**		
Bernese	1	3.2
Afghan hound	2	6.5
Australian Shepherd	2	6.5
Basset hound	2	6.5
Boston Terrier	1	3.2
Coton de Tulear	2	6.5
English Bulldog	2	6.5
French mastiff	1	3.2
Goldendoodle	1	3.2
Husky	2	6.5
Labrador	3	9.7
Mini dachshund	1	3.2
Mixed	11	35.5

In the 31 procedures, the mean ostectomy size was 1.56 ± 0.81 cm, with a range of 0.5–4 cm; all ostectomies were positioned in the distal half of the ulna. The ostectomies accounted for a mean of 12.6 ± 5.7% of the ulnar length. One limb had a fat graft placed intra-operatively. The mean total anesthesia times were 116.3 ± 27.84 min and 179 ± 18.65 min for unilateral and bilateral procedures, respectively. Of 31 limbs, 22 (71%) were bandaged for 2 weeks post-operatively.

Of 31 limbs, 10 (32.3%) experienced complications in the post-operative period. Seven (70%) of these 10 complications were minor, consisting of four cases of incisional erythema and three cases of dermatitis. Two cases of incisional erythema and all dermatitis cases were believed to be bandage induced. The remaining three complications were major; all were full thickness wounds secondary to bandages requiring antibiotic treatment, but no additional surgery was performed. Overall, eight of the total complications were bandage related. Of the 22 (36.4%) bandaged limbs, eight had complications; of the nine (22.2%) unbandaged limbs, two had complications. One-way ANOVA was conducted to determine the effect of anesthesia time on complication type (none, minor, major). The results indicate a non-significant effect, *F*_(2, 25)_ = 1.07, *p* = 0.357. We therefore fail to reject the null hypothesis and conclude that there is no effect of anesthesia time on complication type (none, minor, major). Kruskal-Wallis test was conducted to determine the effect of ostectomy size or age at the time of surgery on complication type (none, minor, major). The results indicate a non-significant effect of ostectomy size, $\chi_{\left(2\right)}^{2}$ = 4.82, *p* = 0.090. The results indicate a non-significant effect of age, $\chi_{\left(2\right)}^{2}$ = 0.32, *p* = 0.854. We therefore fail to reject the null hypothesis and conclude that there is no effect of ostectomy size or age on complication type.

**Figure 4:**
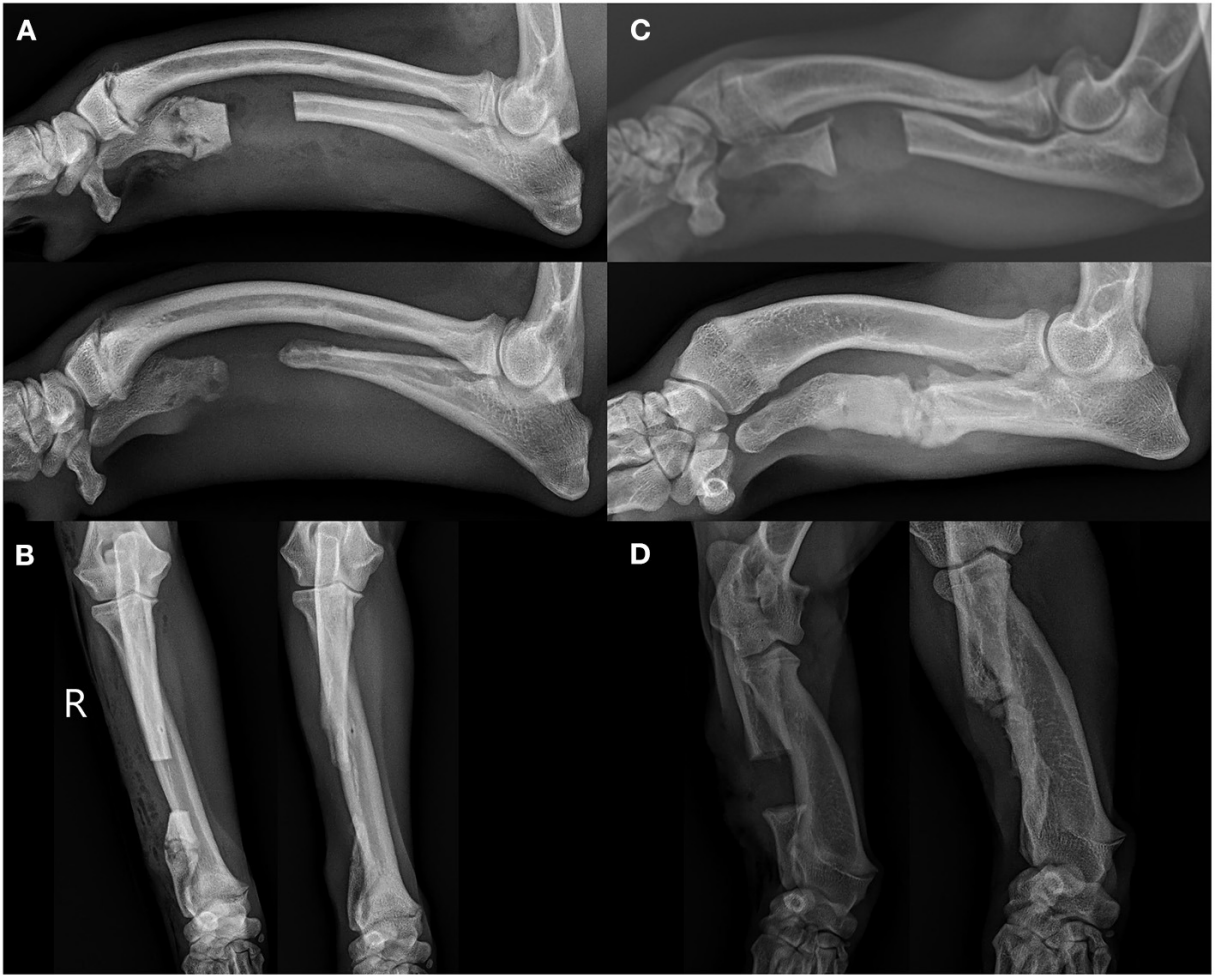
**(A)** Shows the lateral projection of a 7.5 month old Boston terrier at the time of surgery (left) and 8 week follow up (right) showing the absence of a bridging callus at follow up. **(B)** Shows the accompanying post-surgical and follow up craniocaudal projections. **(C)** Shows the lateral projection of a 9 month old mixed breed of equivalent size at the time of surgery and 8 week follow up showing the formation of bridging callus across the ostectomy site. **(D)** Shows the accompanying craniocaudal projections of this mixed breed dog.

**Figure 5:**
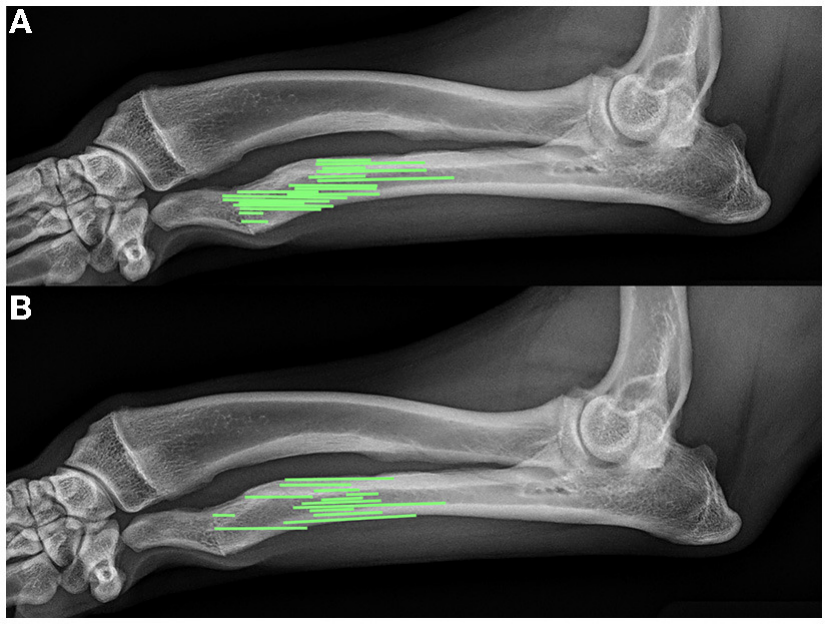
Comparison of relative ostectomy sizes (based on percentage of limb length) and position in patients with **(A)** non-healing ostectomies and **(B)** healing ostectomies. This figure represents the ostectomy percentages and relative locations scaled to a single representative lateral radiograph.

In 16 of 31 (51.6%) elbows, >2 mm radioulnar incongruity was present pre-operatively. Of those limbs, 12 of 16 (75%) had <2 mm radioulnar incongruity at the 8 week follow up. In 7 of the 31 elbows (22.6%), radiographic evidence of elbow degenerative joint disease was present pre-operatively. The same 7 of 31 (22.6%) showed radiographic evidence of degenerative joint disease on follow up radiographs. No degenerative joint disease was observed in the carpus in any patient at the time of surgery or during follow up radiographs. Bridging callus was present at the ulnar ostectomy in 13 of 31 (41.9%) limbs, and the distal radial physes were closed in 20 of the 31 (64.5%) at the time of follow up radiographs. The average age of those animals with a closed physis was 9 ± 1.5 months at follow up. Figure 4 shows representative radiographs of the absence and formation of bridging callus. The mean ostectomy size of limbs with clinical union at follow up was 1.63 ± 0.94 cm (12.03 ± 6.42% of total limb length), compared with 1.51 ± 0.73 cm (13.04 ± 5.27%) in the non-healed limbs; no statistical difference was observed (*p* = 0.69). The position and ostectomy size are demonstrated in Figure 5 as a percentage of a representative lateral radiograph.

Of the 31 treated limbs, 20 (64.5%) showed resolution of grade 1 lameness, and 4 (12.9%) showed a clinical improvement from grade 3 lameness to grade 1. No improvement in lameness was observed in seven patients (22.6%), one of which showed persistent elbow incongruity on post-operative follow up radiographs, and four of which (57.1%) had degenerative joint disease. NSAIDs were used in 10 (43.5%) of the 23 patients prior to surgery which included a total of 14(45.%) of the limbs. Carprofen was used in 7 dogs and meloxicam was used in the remaining 3 patients. At the time of follow-up evaluation, only 2(8.7%) of the patients had continued NSAID usage encompassing 4 (12.9%) limbs. There were no patients in this study group that began NSAID usage following ulnar ostectomy outside of the immediate post-operative period which lasted no longer than 10 days. One of the dogs who remained on NSAIDs had complete resolution of lameness of both limbs while there was no improvement in either limb on the other patient who remained on NSAIDs.

A total of 24 limbs had well positioned frontal plane radiographs and 28 limbs had well positioned sagittal plane radiographs at both pre-operative and follow up time points for measurement of joint orientation angles. The mean pre-operative aLDRA degrees is 78.01 ± 6.79. The mean post-operative aLDRA degrees is 77.34 ± 7.86. The mean pre-operative aCdDRA is 78.05 ± 6.96. The mean post-operative aCdDRA is 80.24 ± 6.78. The mean pre-operative aCdPRA is 86.96 ± 5.95. The mean post-operative aCdPRA is 87.97 ± 8.66. The mean pre-operative theta is 29.06 ± 10.01. The mean post-operative theta is 30.51 ± 11.24. The mean pre-operative procurvatum is 44.12 ± 15.41. The mean post-operative procurvatum is 42.21 ± 17.97. Paired samples t-test was conducted to deterermine whether there is a difference between for pre and post aLDRA, aCdDRA degrees, aCdPRA degrees, theta, and procurvatum degrees. The results indicate non-significant differences between pre- and post-operative aLDRA [*t*_(24)_ = 0.37, *p* = 0.717]; between pre-operative and post-operative aCdDRA [*t*_(28)_ = −1.46, *p* = 0.156]; between pre- and post-operative aCdPRA [*t*_(28)_ = −0.88, *p* = 0.388]; between pre- and post-operative theta [*t*_(28)_ = −1.29, *p* = 0.209]; and between pre- and post-operative procurvatum [*t*_(28)_ = 0.77, *p* = 0.447]. These numbers are summarized in Table 4. We therefore fail to reject the null hypothesis and conclude that there is no difference between for pre and post aLDRA, aCdDRA degrees, aCdPRA degrees, Theta, procurvatum degrees. Repeated measures ANOVA was conducted to determine the effect of time follow up time and age on aLDRA, aCdDRA, aCdPRA, theta, and procurvatum. The results indicate a non-significant main effect of follow up time, *F*_(1, 24)_ = 0.51; *p* = 0.483, partial eta squared = 0.026 and a non-significant interaction effect of age on aLDRA, *F*_(1, 24)_ = 0.43; *p* = 0.521, partial eta squared = 0.022. The results indicate a non-significant main effect of follow up time, *F*_(1, 28)_ = 1.07; *p* = 0.310, partial eta squared = 0.040 and non-significant interaction effect of age on aCdDRA, *F*_(1, 28)_ = 1.07; *p* = 0.310, partial eta squared = 0.040. The results indicate a non-significant main effect of follow up time, *F*_(1, 28)_ = 1.13; *p* = 0.297, partial eta squared = 0.042 and a non-significant interaction effect of age on aCdPRA, *F*_(1, 28)_ = 0.81; *p* = 0.376, partial eta squared = 0.030. The results indicate a non-significant main effect of follow up time on theta, *F*_(1, 28)_ = 0.001; *p* = 0.972, partial eta squared = 0.000 and a non-significant interaction effect of age on theta, *F*_(1, 28)_ = 0.06; *p* = 0.815, partial eta squared = 0.002. The results indicate a non-significant main effect of time on procurvatum, *F*_(1, 28)_ = 0.013; *p* = 0.911, partial eta squared = 0.000 and a non-significant interaction effect of age on procurvatum, *F*_(1, 28)_ = 0.08; *p* = 0.783, partial eta squared = 0.003. We therefore fail to reject the null hypothesis and conclude that there is no effect of follow up time and age on all measured joint orientation angles.

**Table 4:** Paired samples *t*-test results including the number of limbs evaluated (N), the arithmetic mean, standard deviation (SD), the *t*-value (*t*), and *p*-value (*p*).

		***N***	**Mean**	**SD**	***t***	***p***
Pair 1	Pre aLDRA	24	78.01	6.79	0.37	0.717
	Post aLDRA	24	77.34	7.86		
Pair 2	Pre aCdDRA	28	78.05	6.96	−1.46	0.156
	Post aCdDRA	28	80.24	6.78		
Pair 3	Pre aCdPRA	28	86.96	5.95	−0.88	0.388
	Post aCdPRA	28	87.97	8.66		
Pair 4	Pre Theta	28	29.06	10.01	−1.29	0.209
	Post Theta	28	30.51	11.24		
Pair 5	Pre Procurvatum	28	44.12	15.41	0.77	0.447
	Post Procurvatum	28	42.21	17.97		

## Discussion

According to the results of this study, distal ulnar ostectomy was associated with few major or catastrophic complications but had not significantly corrected angulation of the antebrachium by the post-operative follow-up evaluation.

In this study group, animals with bilateral disease and unilateral disease were treated as independent limbs. These groups were first analyzed independently with no significant difference regardless of the presence of unilateral or bilateral disease. It is reasonable to assume the biomechanics of the limbs with bilateral disease differ compared to that of dogs with unilateral disease. The groups were first analyzed independently and no difference was found between the two groups. In dogs with unilateral elbow disease, a component of weight shifting is the transition of weight to the contralateral limb based on force plate analysis (40). There are no studies to my knowledge that attempt to characterize the extent of caudal weight shifting in dogs with and without bilateral forelimb disease. The degree of this weight bearing will likely change with the type and severity of disease. For example, in our study it is reasonable to assume in dogs with unilateral disease that there will be an alteration in biomechanics, weight bearing, and limb angulation compared to bilateral disease as the propensity for weight shifting to the contralateral limb will be decreased and may be replaced with either increased weight bearing on the affected limbs or with a caudal shift to weight bearing in the pelvic limbs. It is very possible there will be both; however, we do not know the magnitude of these weight shifting during ambulation and during the stance phase nor do we know how the alteration in weight shifting will alter loading at the stance phase and during ambulation. Additionally, any concurrent orthopedic disease, such hip dysplasia, can alter loading in dogs with unilateral or bilateral disease. Due to the lack of quantitative gait analysis, the inability to adequately predict how bilateral disease will affect limb loading, and the lack of statistical significance and small sample sizes obtained when treating bilateral disease independently, each limb was treated independently regardless of whether their treatment was unilateral or bilateral.

Before evaluating the effect of an ulnar ostectomy particularly over such a short time span, we have to understand the growth progression of juvenile dogs and angular limb deformities. Growth rates and overall limb length is expected to vary greatly between large and small dogs. Using the tibia as a model, long bone growth occurs as a combination of saltation and stasis with the majority of the growth occurring early in the first year of life and slowing down gradually throughout that time frame (41). This tibial model consisted entirely of Labradors however studies in varying species have shown the rates of the same bone will change in overall length however they will usually maintain a similar rate during these times frames (42). While it is important to understand normal growth rates, the timing and rates of deformity correction are essential to evaluating the potential for deformity correction in this study given the short 8 week follow up time frame. Ramadan et al. evaluated 58 cases of premature distal ulnar physeal closure in 1978 (5). Of the 58 dogs in that study, the majority were large breed dogs who were 5–7 months old at the time of diagnosis. 50 of the 58 dogs were surgically treated through a variety of procedures. 39 of the dogs treated for carpal valgus in the study using radial physeal stapling. The application of this compression corrected the deformity in an average of 45 days with some limbs correcting in as little as 3 weeks. Additionally, Ramadan observed removal of the physeal staple resulted in a fairly rapid return of physeal longitudinal growth which allows recovery of any length discrepancy that had occurred. Ramadan's observations show that while the 8 week follow up is short, it is reasonable to think a change in limb angulation can occur during this time frame. Ulnar ostectomy was not performed frequently in this study and like other studies it was combined with radial stapling.

In contrast to previous literature, our study focuses on ulnar ostectomy as a sole means of treatment for premature ulnar physeal closure. Bridging callus of the ostectomy at the follow up radiographs averaging 7.5 weeks post-surgery occurred in ~40% of patients. Although radiographic or clinical outcomes were not found to be affected, the formation of bridging callus may be associated with worsening of an angular limb deformity depending on the degree of growth still present at the physis. A total of 5 of 13 (38%) limbs with a healed ostectomy at follow up had open radial physes, which could allow for further progression of angular limb deformity when performed in dogs with more growth capacity. Although the long-term outcomes are currently unclear in the patients evaluated in this study, preventing bridging callus would be ideal for all patients, particularly younger patients with high growth potential at the time of surgery. Several controllable factors that may prevent premature healing include ostectomy size, periosteum treatment, and fat graft usage. Throughout this study, a large variation in ostectomy size was observed in all patients, but no significant difference was observed between clinically healed limbs and those that did not heal. Ostectomy size (as a percentage of total ulnar length) and relative position (Figure 5) did not have an impact on bone healing. Each ostectomy was positioned between the mid ulna and distal ulnar physis. Size of the ostectomy and ostectomy location varied by surgeon. Overall the procedures were performed by seven different surgeons which explains the variance in size and location. Ideally a single ostectomy size and location or a series of ostectomy sizes and locations would be preferable in the study process to evaluate the effect of either size or location or both and it minimizes variability in the analysis of outcomes. Most of the veterinary literature in critical bone defects has focused on overcoming critical bone defects; however, very little literature has defined what truly constitutes a critical defect. Kraus et al. have documented the critical defect nature of a 2.1 cm femoral gap with periosteum removed (43). According to this definition, only five of the patients treated in this study met the criteria for a critical defect, two of which developed a bridging callus by 8 weeks post-surgery, thus suggesting that this definition of critical defects does not apply to the ulna in the same manner as the femur. The high frequency of bridging callus in limbs prompts questions regarding the need for further focus on surgical guidelines aside from strict ostectomy size to prevent bridging bone formation. In human research, a critical bone defect usually consists of bone loss of >1–2 cm and >50% loss of the circumference of the bone (44–46). Ideally, we in the veterinary field should at least aim to meet these minimal criteria. Critical bone defects will likely have a variety of confounding factores including physical separation, anatomic location, microenvironment, circumference of the defect, and loss of vascular supply (44, 47).

Aside from critical size of the defect, multiple factors can affect ostectomy healing. The periosteum is an integral component of bone healing (48). As shown by Huh et al. in the canine mandible, the critical defect size is dependent on the presence or absence of the periosteum (49). In this study, 16 dogs had mandibular defects created with sizes ranging from 5 to 20 mm without periosteum and 30–60 mm with intact periosteum. The critical mandibular defect without periosteum was 15 mm, compared with 50 mm when periosteum was preserved. To minimize the chance of bridging bone callus, careful attention must be paid to optimizing the removal of an appropriately sized ostectomy segment as well as ensuring that the maximal amount of periosteum is excised. An additional procedure to minimize bone healing is the use of autogenous fat grafts. The use of an autogenous fat graft was originally based on Erich Lexer's finding in the early 1900s that fat maintained its structure when transplanted to new locations. Years later, Osterman's 1972 study detailed fat graft placement to prevent closure and healing in a femoral partial premature epiphyseal closure rabbit model (50). The biologic behavior of a premature physeal closure rabbit model of a single bone had been assumed to act identically to the radius and ulna. Elliot Craig showed that autogenous fat grafts were sufficient to delay healing of distal ulnar ostectomies in a case series of 10 patients (51). In that study, Craig also completely removed the periosteum associated with a 2 cm ostectomy segment, which theoretically should have been a critical bone defect. Only one patient in this study received an autogenous fat graft. The goal of both autogenous fat graft or removing a critical bone defect is to minimize the risk of developing bridging callus. In the event in which an autogenous fat graft is used, minimizing graft trauma and using an appropriately sized graft are crucial for success.

This study did not show a significant difference in frontal and sagittal plane joint orientation lines between pre-operative and follow up radiographs. Hueter-Volkmann proposes longitudinal bone growth during development is slowed by mechanical compressive forces and accelerated by tensile forces. The timing of these effects in endochondral ossification and longitudinal bone growth is unclear. The overall timing of skeletal maturity varies between species and in the case of canines between size of animals. While the growth rate of different bones and different species vary greatly, the response to compressive forces appears to be more consistent. Stokes et al. showed static compressive forces applies to the proximal tibia and caudal vertebra of rats, rabbits, and calves at two different ages were consistent regardless of bone or species. The growth-rate sensitivity to stress in this study averaged 17.1% per 0.1MPa with both tension and compression despite there being a large variation in overall growth rates based on the bone and species (42). In the case of dogs with premature closure of the ulnar physis, the abnormal ulnar physis essentially provides a compressive force similar in the manner which a physeal staple does. This compressive force slows the growth of the caudolateral radial physis leading to the deformities seen in these patients: valgus in the frontal plane and procurvatum in the sagittal plane. The deformity extent is dependent on the growth potential at the time of the application of compressive forces as well as the extent and duration of the application of the compressive forces. Ulnar ostectomy should halt the statically applied compressive force which is slowing growth. Removal of this external compressive force should allow the radial growth to return to normal rates which should allow equivalent growth of the sides of the radial physis assuming no other external forces alter longitudinal growth rates. Application of the Hueter-Volkmann theory suggests that deformity progression should continue as the compressive forces of the radius become more caudolaterally focused as the deformity progressives leading to a theoretical progression of the limb deformity. It has typically been thought that longitudinal growth is a continuous process which would support the idea that limb deformity progression occurs due to abnormal weight bearing and compressive loading of the physis however this is not supported by the findings of this study. There are several reasons this could have occurred. This could be due to the age and follow up length in these patients. It is possible that we did not see progression because of the short time frame in this study however 65% of the animals in this study had radiographically closed radial physes at the time of follow-up which would not allow further alterations in longitudinal growth. The average age of the remaining 11 was ~9 months of age which allows some continued growth which may allow further progression. Another option is alterations in limb positioning and weight bearing altered the mechanical axis of the limb enough to shield from progression of limb deformity however this seems unlikely as many of the dogs had improved lameness during gait analysis. Alternatively, Noonan et al. evaluated bone growth in an ovine model to evaluate growing pains in children. They implanted microtransducers in immature lambs and measure bone length every 167 s for 21–25 days showing 90% of longitudinal limb growth occurred during recumbency and virtually no growth occurred during standing or locomotion (52). In this case, ulnar ostectomy alone would remove the compressive force on the radial epiphysis and result in uniformity of radial physeal growth rates regardless of the loads applied during ambulation. This would support the lack of progression of limb deformity following ulnar ostectomy found in this study. It has also been suggested Wolff's law becomes the predominant guiding principle in older animals. In rat models, applied compressive force resulted in vertebral body thickening rather than wedge formation in older rats (19). This would also explain a lack of progression of angulation but result instead in cortical thickening. This finding would agree with the radiographic evaluation seen here in regards to joint orientation however cortical thickening was not able to be evaluated due to the modality used. There is no difference in procurvatum or valgus in patients between pre-surgical and follow up time frames however ulnar ostectomy has been shown to load the caudal cortex of the radius leading to new bone deposition in a sheep model (53). This new bone deposition has the potential to alter the appearance of the limb radiographically however the necessary adaptive time of this process in dogs is unknown.

An earlier study by Morgan et al., examining the effects of ulnar ostectomy, has evaluated 24 dogs with corrective osteotomies and ultimately evaluated 12 dogs with partial ulnar ostectomies, and 60% of the dogs had good to excellent function following surgical treatment (30). Of the 12 dogs and 16 treated limbs in Morgan's study, a significant difference in frontal angles before and after surgery was observed. Nine patient radiographs were available for pre-operative and follow up frontal plane measurement. Sagittal plane measurements were not evaluated in Morgan's study. The smaller numbers of treated limbs in Morgan's study might have led to an improvement in frontal plane angulation, which was not seen in the larger number of treated limbs seen in this study. Two of nine treated limbs in Morgan's study with paired pre- and post-operative measurements showed an improvement exceeding 10°, whereas the other improvements were more modest. The mean age of Morgan's patients was 7.93 months; however, the follow up time frame was at least 6 months. Continued growth with an untethered ulna might potentially lead to significant changes in angulation. The breeds of the treated dogs in Morgan's study are undescribed; therefore, continued growth in younger or large breed dogs might have accounted for the significant improvements in a several dogs in that study. In addition, unidentified injuries to the distal radial physis might have been present in some patients in Morgan's study, or changes in frontal plane angulation in the present study might have been mitigated by premature tethering of the distal radius due to fibrosis or bridging callus at the ulnar ostectomy site. Morgan showed that 60% of limbs had good to excellent outcomes. In this study, 65% of canines undergoing ulnar ostectomy showed complete resolution of clinical lameness, on the basis of orthopedic examination, 15% showed improvement without complete resolution, and 20% showed no clinical improvement. Given the complex nature of the limb deformities as well as the potential for additional cubital joint injuries (21, 54), the finding of persistent lameness in some patients is unsurprising. Complications were not evaluated in Morgan's study.

An overall complication rate of 32.2% was found, 80% of which were bandage related. Post-operative bandaging was performed in 70.9% of dogs. As the bandages did not include any splinting, the bandage was intended to apply compression to the operated limb. Full thickness wounds were the most common major complications while erythema and dermatitis. The higher than normal bandage related complications can in part be attributed to the procedure being performed on young, likely active juvenile animals. While these complications did not significantly alter overall outcomes, several steps could be taken to minimize them. These include more frequent bandage changes to ensure wound exudate and external moisture as less likely to contribute to dermatitis and increased sedation usage and patient compliance to minimize bandage slippage and any associated wounds. Although a large discrepancy was found between the number of bandaged and unbandaged limbs, only 22% of the unbandaged limbs had complications, compared with 36% of the bandaged limbs. Neither seromas nor significant limb swelling were reported in the population of dogs without post-operative bandages. Because of the lack of clear evidence of support, low frequency of seroma formation, and high frequency of bandage related complications, bandage use should be carefully considered on a patient-by-patient basis.

Overall our study documents the complications seen with ulnar ostectomy in juvenile dogs. It also documents a lack of correction and progression of angular limb deformities over the course of 2 months following ulnar ostectomy. While not every dog has a closed radial physis, the majority of their longitudinal bone growth should be complete in the animals whose physis is not closed at the time. The clinical outcomes are similar to those historically seen in the literature.

## Limitations

This study is retrospective in nature and as such we are limited to the information that is available in the record without the ability to acquire additional information such as concurrent disease processes such as hip dysplasia, additional time points for follow up, or an appropriate identical control group. In this particular case, one of the major limitations is the lack of internal controls. Radiographs of the contralateral limb for patients with unilateral disease were not available. Multiple surgeons performed the procedure over the 7-year time span in which cases were enrolled, thus partially explaining the variation in ostectomy size. The use of radiographs to quantify and classify deformities has been shown to be repeatable; however, single projections were evaluated for each limb with the center of the radius being the focal point of the image beam. This minimizes radiographic parallax as much as possible by minimizing the angle of the beam in relation to the object as much as possible. While it may change overall size and length measurements, it is unclear how much parallax would alter angle measurements. If future studies are to be performed, CT imaging would be preferred to evaluate elbow joint space as well as limb torsion and would also allow evaluation of focal cortical thickening due to abnormal force distribution. The use of CT also removes parallax as a major concern. If CT were not performed, radiographs centered over the length of the radius as well as at the carpus and elbow would be ideal to minimize any variable related to radiograph beam alignment. Ideally, arthroscopic evaluation of the joint would have been performed as well, given its ability to evaluate the joint for congruence at multiple locations and to reveal cartilage damage and the presence of a fragmented medial coronoid process which can be seen in conjunction with elbow incongruency and may lead to continued lameness. Another limitation is single evaluator performed all radiographic measurements. Although the evaluator was blinded to the status of the distal ulnar physis, blinding the evaluator to whether the distal radial physis was closed was impossible; therefore, the observer might have been able to identify the time point being measured. These images were compared with pre-operative films rather than normal limb radiographs. An additional limitation is the small number of evaluated patients as well as the subjective measurements of lameness improvement.

A larger prospective clinical study with CT imaging at both initial evaluation and serial follow up until physeal closure, arthroscopy, objective lameness measurements such as force plate or temporo-spatial kinematic analysis, and more frequent follow ups to follow bone healing and deformity progression would provide more information and enable more accurate quantification of the limb deformity and the change in angulation following surgery.

Despite these limitations, this retrospective study provides important previously unknown information about the frequency of bridging callus formation after distal ulnar ostectomy as well as clinical improvement in dogs undergoing distal ulnar ostectomy for premature closure of the distal ulnar physis. This study also confirms that ulnar ostectomy can prevent the progression of carpal valgus but does not enable a significant correction of the radial deformity present at the time of surgery. Further studies may also investigate the importance of fat graft usage and periosteum resection on bony healing as well as the requirements for creating the critical defect needed to prevent bridging callus and subsequent repeat tethering of the distal ulna.

Previous studies have suggested possible improvement in antebrachial angulation that occurs with distal ulnar ostectomy. This study rejected the null hypothesis that distal ulnar ostectomy would improve joint orientation lines. Ulnar ostectomy prevented significant progression of angular limb deformities secondary to premature ulnar physeal closure but did not lead to improvement at the end of this follow up period. This procedure also appeared to have few major complications, which were most commonly associated with the application of a modified Robert-Jones bandage.

## Data availability statement

The raw data supporting the conclusions of this article will be made available by the authors, without undue reservation.

## Author contributions

The author confirms being the sole contributor of this work and has approved it for publication.

## Conflict of interest

The author declares that the research was conducted in the absence of any commercial or financial relationships that could be construed as a potential conflict of interest.

## Publisher's note

All claims expressed in this article are solely those of the authors and do not necessarily represent those of their affiliated organizations, or those of the publisher, the editors and the reviewers. Any product that may be evaluated in this article, or claim that may be made by its manufacturer, is not guaranteed or endorsed by the publisher.
